# Epidemiology of Nontuberculous Mycobacteria in Patients without HIV Infection, New York City

**DOI:** 10.3201/eid1403.061143

**Published:** 2008-03

**Authors:** Ethan E. Bodle, Jennifer A. Cunningham, Phyllis Della-Latta, Neil W. Schluger, Lisa Saiman

**Affiliations:** * Columbia University, New York, New York, USA

**Keywords:** Nontuberculous Mycobacteria in Patients without HIV, New York, Atypical mycobacterium infection, Mycobacterium avium complex, Mycobacterium abscessus, Mycobacterium kansasii, Mycobacterium fortuitum, Mycobacterium marinum, respiratory tract, epidemiology, incidence, research

## Abstract

We reviewed medical records of patients without known HIV and with positive cultures for nontuberculous mycobacteria (NTM) isolated during 2000–2003 from 1 large hospital in New York, New York. Overall, 505 patients had positive NTM cultures; 119 (24%) met the criteria for NTM disease. The difference between demographic characteristics of case-patients in our study (66% female, 61% white, and 59% >60 years of age) and those of the base population as determined by regional census data was statistically significant. Estimated incidences for positive cultures, all disease, and respiratory tract disease were 17.7, 2.7, and 2.0 per 100,000 persons, respectively. More patients with rapidly growing mycobacteria (61%), *Mycobacterium kansasii* (70%), or *M. marinum* (100%) met criteria for disease than did patients with *M. avium* complex (MAC) (27%, (p<0.01). NTM disease in patients without HIV is increasing. Laboratory-based surveillance may be useful for detecting non-MAC and non–respiratory tract disease.

## Abstract 2

The incidence appears to be increasing.

Although the pathogenic potential of nontuberculous mycobacteria (NTM) was reported throughout the 20th century, widespread appreciation of the clinical syndromes caused by NTM began during the 1980s in association with the AIDS pandemic and the consequent dramatic increase in disseminated *Mycobacterium avium* complex (MAC) infections (*1**,**2*). However, the epidemiology of NTM disease in patients without HIV infection remains somewhat difficult to determine. NTM disease is relatively uncommon (*3*); it is not a reportable health event, and environmental exposure varies greatly by geographic region (*1**,**4*). Further, clinically insignificant colonization or contamination can be difficult to distinguish from true disease, which can render laboratory-based surveillance potentially inaccurate (*5*), and the risk factors for disease have not yet been fully defined.

To expand our understanding of the epidemiology of NTM, we reviewed the demographic and clinical characteristics of patients without known HIV infection who had positive cultures for NTM from 2000–2003. We sought to determine the incidence of NTM disease and colonization, the risk factors for NTM disease, and the species of mycobacteria associated with different clinical syndromes at our urban medical center.

## Methods

### Study Design and Site

We conducted a retrospective study of patients without known HIV infection and with positive cultures for NTM obtained during 2000–2003 at Columbia University Medical Center (CUMC), New York-Presbyterian Hospital, the only medical center in northern Manhattan. The study was approved by the Institutional Review Board of Columbia University.

### Study Patients

Study patients had positive cultures for NTM and no laboratory evidence of HIV infection. Our mycobacteriology laboratory compiled the medical record numbers of patients with positive NTM cultures from 2000 through 2003. To maintain privacy regarding HIV status, the list was electronically purged of the names of patients with positive HIV serologic test results, patients with HIV viral load, and patients who had had genotyping studies performed. Patients identified in clinical notes as HIV infected were excluded.

### Data Collection

Demographic characteristics, coexisting medical illnesses, and results of computed tomography (CT) studies of the chest and mycobacteriologic studies were collected from electronic medical records. These records were generally complete for demographic characteristics and clinical microbiology laboratory, surgical, and radiographic reports but sometimes lacked progress notes or treatment records, which were often written by hand in bedside charts. Electronic medical records were considered adequate to assess risk factors if the clinical notes (progress notes, consultation notes, discharge summaries) documented the medical history, coexisting illnesses, and medication regimens, including use of antimycobacterial agents.

### Case Definitions of NTM

Patients with blood cultures or tissue biopsy specimens positive for NTM were considered to have NTM disease. Patients with positive respiratory tract cultures were considered to have pulmonary disease if they met the following American Thoracic Society (ATS) guidelines (*6*): chest CT scan performed within 6 months of an NTM-positive culture demonstrating infiltrates, nodules, cavities, bronchiectasis, or tree-in-bud formations and >3 NTM-positive respiratory cultures; 2 positive cultures with >1 positive acid-fast smear; or 1 positive culture with moderate, many, or heavy acid-fast bacilli noted on smear. Patients were considered not to have disease if NTM had been isolated from stool or urine or if a nonpathogenic NTM species (e.g., *M. gordonae* or *M. gastri*) had been isolated but symptoms attributed to another etiology.

### Estimated Incidence of NTM Disease

We estimated the annual incidence of NTM disease by using previously described methods (*7*). Since the total population at risk was unknown, we calculated a rough incidence estimate by studying only those patients with positive NTM cultures who resided in the geographic area, which was closer to CUMC than to any other New York City hospital. Residents in this area, which encompassed 5 ZIP codes, were assumed to have the highest probability of receiving medical care at CUMC, and in fact, these ZIP codes were the most commonly noted among patients at CUMC. The number of cases of NTM disease diagnosed in this geographic subset per year was then divided by the 2000 US Census population for the same area to calculate the incidence estimate (*8*). The population was adjusted downward by 1.5% based on current estimates of the HIV/AIDS prevalence in New York City.

### Demographic and Geographic Analysis

The demographic characteristics of the study patients were compared with those of the New York Public Health Department (NYPH) catchment population using 2000 US Census data (*8*). We calculated the distribution of sex and race in the catchment population from weighted census tract data. To evaluate the potential role of environmental exposure, we compared the distribution of the residence ZIP codes of patients with NTM isolates to the ZIP codes of all CUMC patients.

### Statistical Analysis

Associations and confidence intervals (CIs) were calculated with SAS 9.1 (SAS Institute Inc., Cary, NC, USA) and EpiInfo 3.3.2 (Centers for Disease Control and Prevention, Atlanta, GA, USA). Single-proportion CIs were derived from the binomial distribution with continuity correction. We calculated CIs for the incidence estimate by using the formula provided by the National Center for Health Statistics (*9*). Median ages were compared by using the Mann-Whitney-Wilcoxon test. Univariate and multivariate associations between clinical and mycobacteriology data used Fisher exact test and logistic regression, respectively. Reported CIs and 2-tailed p values were for the 95% confidence level; p values are given without correction for multiple comparisons.

## Results

**Figure 1:**
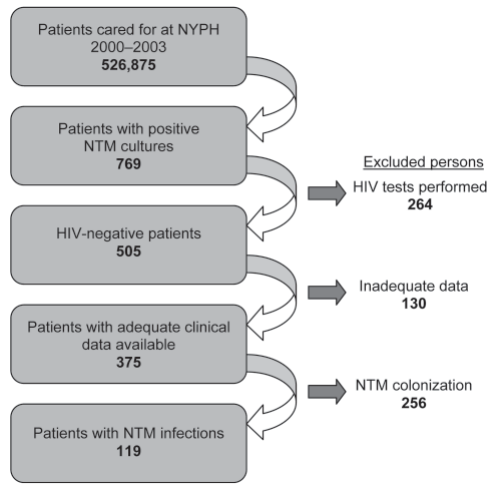
Flowchart of patient selection for cases of nontuberculous mycobacteria (NTM) colonization and NTM disease among patients without HIV infection, New York–Presbyterian Hospital (NYPH), Columbia University Medical Center, 2000–2003.

During the 4-year study period, the clinical microbiology laboratory identified 769 patients with at least 1 positive NTM culture. Of these, 264 were excluded from further analysis by electronic purge of HIV-infected patients as previously described (Figure 1). The remaining 505 study patients had 820 positive NTM cultures; 282 (56%) were hospitalized when their first positive NTM culture was obtained. MAC and the rapidly growing mycobacteria (RGM) species were most common, isolated from 84% (n = 422) and 9% (n = 45) of patients, respectively (Table 1).

**Table 1:** HIV-negative patients with positive nontuberculous mycobacteria cultures and disease, New York–Presbyterian Hospital, Columbia University Medical Center, New York, New York, 2000–2003

NTM species*	No. positive cultures	Adequate data to assess case status†	No. patients with disease (%)‡
All species	505	375	119 (32)
*Mycobacterium avium* (MAC) complex	422	297	79 (27)
Rapidly growing mycobacteria‡	45	41	25 (61)
*M. abscessus*	14	13	11 (85)
*M. chelonae*	15	13	4 (31)
*M. fortuitum*	16	15	10 (67)
*M. gordonae*	25	6	0
*M. kansasii ‡*	12	10	7 (70)
*M. marinum ‡*	4	4	4 (100)
*M. scrofulaceum*	5	4	0
*M. xenopi*	13	9	5 (56)

*M. flavescens, *M. gastri*, *M. haemophilum*, and *M. neoaurum* were isolated once each. †Patients with adequate clinical, radiographic, and mycobacteriologic data to assess case status. ‡Greater proportion of rapidly growing mycobacteria, *M. kansasii,* and *M. marinum* caused nontuberculous mycobacteria (NTM) disease when compared with MAC (p<0.01).

### Proportion of Patients with NTM Disease

Of the 505 study patients with NTM-positive cultures, 375 (74%) had adequate clinical data to determine disease status. In all, 119 (32%) of 375 were considered to have NTM disease (Table 1, Figure 1). A significantly higher proportion of patients with RGM (61%), *M. kansasii* (70%), or *M. marinum* (100%) isolates were categorized with disease compared with those with MAC (27%) isolates (p<0.01).

### Body Site and NTM Species

#### Respiratory Tract

Although only 24% (81/344) of patients with NTM-positive cultures from the respiratory tract met ATS criteria for NTM disease, 68% of cases of disease occurred in the respiratory tract (Table 2). However, MAC, RGM, *M. xenopi,* and *M. kansasii* caused 80%, 9%, 6%, and 5% of these cases, respectively. No NTM species predicted disease; although a higher proportion of patients with *M. kansasii* (57%) and *M. xenopi* (44%) isolates were categorized with disease compared with MAC (25%); these differences were not statistically significant (p = 0.08 and 0.24, respectively). No patient with *M. gordonae,* *M. flavescens*, or *M. scrofulaceum* isolates met ATS criteria for disease.

**Table 2:** Site of disease and species of nontuberculous mycobacteria, New York–Presbyterian Hospital, Columbia University Medical Center, New York, New York, 2000–2003*

Site of disease	No. patients with MAC infection	No. patients with RGM infection	No. patients with other species infections	Total no. patients (%)
Respiratory tract	65	7	9	81 (68.1)
Skin and soft tissue, nonsurgical	2	4	6	12 (10.1)
Surgical sites	0	7	2	9 (7.6)
Bloodstream	2	4	1	7 (5.9)
Lymph node	5	1	0	6 (5.0)
Disseminated	2	0	0	2 (1.7)
Central nervous system	0	1	0	1 (0.8)
Gastrointestinal tract	0	0	1	1 (0.8)
All body sites	76	24	19	119 (100)

*MAC, *Mycobacterium avium* complex; RGM, rapidly growing mycobacteria.

#### Skin, Soft Tissue, and Surgical Wounds

Skin and soft tissue sites were the second most common sites of disease and occurred in 21 (18%) patients. RGM caused 4 (33%) of 12 nonsurgical skin and soft tissue infections and 7 (78%) of 9 surgical wound infections. Seven of the latter were associated with cosmetic procedures; 4 had been performed in the Dominican Republic, 2 in Ecuador, and 1 in the United States. All 4 cases of *M. marinum* infection occurred in the upper extremities.

#### Bloodstream Infections and Disseminated Disease

Seven patients had bloodstream infections (5 with RGM and 2 with MAC). Two additional subjects had positive blood cultures (both with MAC) and other infected body sites and, thus, were categorized with disseminated disease.

#### Gastrointestinal (GI) Tract Isolates

Of the 11 patients with MAC cultured from the GI tract, 7 had adequate clinical information to assess disease status. Two (29%) of 7 had disseminated disease as described above, and 5 had no clinical signs or symptoms of infection.

#### Urine Isolates

No patients had NTM disease of the urinary tract. One patient had 4 urine cultures positive for MAC but was not categorized as having NTM disease because no symptoms of urinary tract infection and no treatment with antimycobacterial agents had been documented.

### Estimated Incidence of NTM Disease

Data from the 2000 US Census showed that 276,032 people resided within 5 ZIP codes that are closer to our medical center than any other hospital. During the study period, 37% of the 536,875 patients cared for at CUMC listed their home addresses within these 5 ZIP codes. Adjusted for the HIV prevalence rate of ≈1.5% in New York City, the base HIV-negative population was 271,892. Overall, 192 (38%) of 505 patients with positive cultures for NTM and 29 (24%) of 119 patients with NTM disease resided in this same area. Thus, the estimated annual incidences of patients with positive NTM cultures in the area defined by these 5 ZIP codes, NTM disease (inclusive of the respiratory tract), and NTM disease specifically of the respiratory tract were 17.7 (95% CI 15.2–20.2), 2.7 (95% CI 1.8–3.8), and 2.0 (95% CI 1.3–3.1) cases per 100,000 persons, respectively.

### Demographic Characteristics

#### Sex

**Figure 2:**
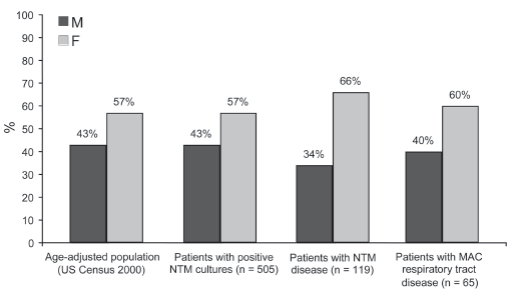
Distribution by sex of patients with positive nontuberculous mycobacteria (NTM) cultures, NTM disease, and disease of the respiratory tract caused by *Mycobacterium avium* complex (MAC), New York–Presbyterian Hospital, Columbia University Medical Center, 2000–2003, compared with age-adjusted base population from 2000 US Census data.

By adjusting 2000 US Census data for age, the expected proportion of women in the base population was 57% (Figure 2). The same proportion was observed in patients with NTM-positive cultures (57.0%, 95% CI = 52.6%–61.4%). In contrast, patients with NTM disease were significantly more likely to be female than were those in the base population (66.4%, p = 0.04). Among those with disease of the respiratory tract caused by MAC, the distribution of patients by sex was similar to that of the base population (60.0% female, p = 0.71).

#### Race and Ethnicity

**Figure 3:**
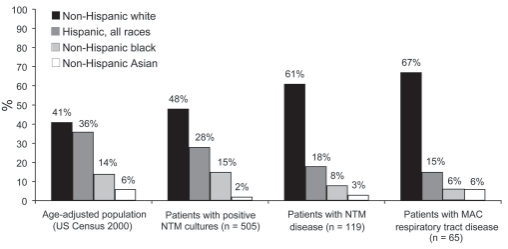
Distribution by race of patients with positive nontuberculous mycobacteria (NTM) cultures, NTM disease, and disease of the respiratory tract caused by *Mycobacterium avium* complex (MAC), New York–Presbyterian Hospital, Columbia University Medical Center, 2000–2003, compared with age-adjusted base population from 2000 US Census data.

The overall distribution of race and ethnicity was significantly different for patients with positive NTM cultures (p<0.01) or disease (p<0.001) when compared with the age-adjusted base population (Figure 3). A greater proportion of patients with NTM disease were white and fewer were Hispanic. Similarly, patients with NTM disease were more likely to be white than patients with a positive culture (61% vs. 48%, p = 0.008).

#### Age

The median age of the study patients with positive NTM cultures was 66 years. Most (59%, n = 70) patients with disease were >60 years of age; only 8% (n = 9) were children <15 years of age. Patients with MAC disease were older than those with RGM disease (68 vs. 53 years of age, respectively, p<0.01). The median ages of patients with disease of the respiratory tract caused by different species were similar (71 years vs. 69 years of age for MAC and RGM, respectively); patients with nonpulmonary disease caused by MAC were substantially younger than those with nonpulmonary disease caused by RGM (11 vs. 41 years of age, respectively).

### Geographic Distribution of Patients

The ZIP codes of patients with positive NTM cultures were compared with those of all patients registered at CUMC. Patients with positive cultures were less likely to live in northern Manhattan within 3 miles of the medical center than were the hospital’s overall patient population (OR 0.72, p<0.001). In contrast, substantially more patients with positive cultures resided in the northwestern area of the Bronx (OR 2.17, p<0.001) or in Staten Island (OR 2.25, p<0.001).

### Coexisting Illness and Concomitant Medications

At least 1 coexisting illness or concomitant medication considered to be a potential risk factor for NTM disease was noted for 73% of patients who fulfilled the study case definitions for disease. Ninety-four (79%) of 119 patients with NTM disease had adequate data to assess their medical histories, and 66% (62/94) had >1 coexisting illness (Table 3). Preexisting lung disease was most common (44%), and patients with respiratory tract disease with NTM were more likely to have preexisting lung disease than patients with disease of other body sites (OR18, 95% CI 4.9–64, p<0.001).

**Table 3:** Comparison of coexisting conditions and concomitant medications with body site of nontuberculous mycobacteria (NTM) disease, New York–Presbyterian Hospital, Columbia University Medical Center, New York, New York, 2000–2003

Site of NTM disease	% Patients with coexisting condition (n = 94)				
	Lung disease	Transplant recipient	Immunocompromised*	Cancer	None
Blood (n = 7)	0	0	33	67	0
Respiratory tract (n = 81)	63	9	16	13	28
Skin and soft tissue, surgical sites (n = 21)	6	19	25	6	62
All†	44	11	18	17	34

Site of NTM disease	% Patients receiving concomitant medications (n = 79)				
	Systemic steroids	Immunosuppressants	Chemotherapeutics	Immunomodulators	None
Blood (n = 7)	50	33	50	17	17
Respiratory tract (n = 81)	26	13	2	4	70
Skin and soft tissue, surgical sites (n = 21)	17	17	8	8	75
All†	25	15	6	5	66

*Defined as diabetes, chronic renal failure and/or rheumatologic disease. †Includes 2 patients with disseminated disease following bone marrow transplantation, 1 patient with central nervous system disease receiving steroids, 1 patient with gastrointestinal disease, and 2 patients with lymph node disease/cancer.

Eighteen percent of patients had >1 immunosuppressive condition (other than transplantation), including diabetes mellitus (14%), chronic renal failure (4%), or rheumatologic disease (5%), and 17% had solid organ or hematologic malignancy. When site of disease (respiratory tract vs. nonrespiratory tract) was adjusted for, patients with MAC disease were more likely to be transplant recipients than were patients with disease caused by other NTM species (OR 7.2, p = 0.01).

For 79 (66%) of 119 patients with NTM disease, data were adequate to assess concomitant medications. Steroids or other immunosuppressive medications were prescribed for 25% and 15% of patients, respectively, within the 6 months before the first positive NTM culture. Although the use of steroids did not predict the site of NTM disease, the use of other immunosuppressive medications was less common in those with disease of the respiratory tract compared with those with disease of nonrespiratory sites (OR 0.30, 95% CI 0.10–0.89, p<0.05). However, when body site was adjusted for, patients with MAC were more likely to have received steroids than were those infected with other species (OR 5.2, 95% CI 1.2–24, p = 0.03). Also, more patients with bloodstream infections received cancer chemotherapeutics than did patients with disease of other body sites (OR 28, 95% CI 3.6–220, p<0.01).

## Discussion

This study is one of the largest recent studies of NTM and reflects the current epidemiology and risk factors for disease and colonization with these microorganisms as assessed in our medical center in northern Manhattan. The rate of NTM disease observed in patients without HIV infection appears to be increasing, but it is difficult to compare studies because different epidemiologic methodols have been used. In a laboratory survey from 1993 to 1996 performed by the Centers for Disease Control and Prevention, the rate of positive NTM cultures was 7.5–8.2 cases per 100,000 persons, compared with our positive culture rate of 17.7 per 100,000 (*5*). The rate of NTM disease derived from several studies conducted through the mid-1990s was estimated to be 2 per 100,000 (*10*). Surveys conducted in Europe estimated the rate of respiratory tract disease with MAC to be 0.2 cases per 100,000, and investigators in the United Kingdom estimated the rate of disease of any body site to be 0.8–3.1 per 100,000 (*11**,**12*). We presented higher estimates of the incidence of NTM respiratory tract disease (2.0 per 100,000) and disease of any body site (2.7 per 100,000), potentially attributable, in part, to improved detection methods. However, our incidence calculation may actually have underestimated the rate of NTM disease if persons who resided in the 5 ZIP codes of interest received care for NTM at another medical facility. Alternatively, had we used a larger geographic region to calculate incidence, we may have compounded the underestimate because additional persons most likely would have sought care at medical facilities other than CUMC. Nevertheless, in the absence of mandatory statewide or nationwide reporting, large institution-based studies can produce the best incidence data.

Variation in the rates of NTM disease and colonization among different populations may also reflect differences in the risk for exposure to environmental mycobacteria. Our data demonstrate geographic variations in the incidence of NTM disease within New York City. Although neighborhood demographics may act as confounding variables, these findings suggest that environmental factors deserve further study. For example, patients residing in the northwestern Bronx had higher rates of disease with NTM; this area receives water from the smaller Croton Reservoir as opposed to the Catskills-Delaware Reservoir that supplies most of New York City (*13*). To test this hypothesis, results of environmental sampling would need to be correlated with cases of human disease (*14**,**15*).

Our study provided an opportunity to study risk factors in a population without referral center bias that can occur in centers specializing in NTM care. The predominance of women among persons with NTM disease is consistent with previous reports (*12**,**16*). For pulmonary disease caused by MAC, the greater proportion of affected women appeared to reflect the higher proportion of women in the older age strata of the base population of NYPH. The correlation of NTM disease with gender did not appear to be attributable to a higher prevalence of concurrent medical conditions or concomitant medication use among women. For example, chronic obstructive pulmonary disease is the most common risk factor for pulmonary disease with NTM, but it is more prevalent among men than women in the United States (89 vs. 61 per 1,000 persons) (*17**,**18*). We speculated that cosmetic surgery could explain, in part, the higher risk of nonrespiratory tract disease in women, although the number of these procedures performed during the study period was unavailable, and cosmetic surgery accounted for only a small number of cases (*19**–**21*). We were also able to uniquely examine the effect of race. As in previous studies, most cases of NTM disease occurred in white persons (*3*), but our base population was unique in having a lower proportion of whites. Furthermore, the higher prevalence of NTM disease noted in whites in our study could not be attributed to cystic fibrosis because only 4 subjects had this medical condition (*22**–**26*).

Only one-third of patients with positive cultures for NTM were categorized with disease. A significantly higher proportion of patients with positive cultures for RGM, *M. kansasii*, or *M. marinum* were considered to have NTM disease than patients with MAC (*27*). Although respiratory tract isolates were most common (*28**,**29*), most reflected colonization or contamination. Thus, laboratory-based surveillance may reasonably estimate the incidence of nonrespiratory tract disease caused by RGM, *M. kansasii,* and *M. marinum* but provide less accurate estimates of the incidence of MAC disease and of respiratory tract disease.

Among the expected risk factors for NTM disease, we found that preexisting pulmonary conditions were most common. However, many cases of NTM disease occurred in patients with concurrent illnesses or medications that were immunosuppressive. Our finding that MAC was the most common pathogen causing posttransplant NTM disease was consistent with results of prior studies (*30**,**31*). Notably, one fourth of patients with NTM disease did not have a known risk factor, which suggests the possibility of a unique genetic susceptibility or environmental exposure.

Our study did have limitations. We used a convenience sample of patients receiving care at our medical center, which introduced potential bias if our sample was not representative of the general population. Our findings may not be applicable to other geographic regions, particularly given the different rates of disease we noted among different areas in New York City. The rare nature of NTM disease makes an accurate measure of the incidence in the population exceptionally difficult. Our incidence rate calculation was a gross estimate and likely an underestimate. Patients residing in the selected base population may have sought care elsewhere; patients with positive cultures and presumptive colonization may have progressed to active disease; and our case-patients were often hospitalized at the time of diagnosis, which suggests limited detection of outpatient cases. In addition, the high proportion of hospitalized case-patients could overestimate coexisting illnesses and concomitant medications. Potential cases of respiratory tract disease could have been missed due to incomplete data, usually a lack of chest CT results. Cultures or radiographic imaging may have been performed at other medical facilities, which could have resulted in misclassification of disease status. Racial differences could reflect, in part, differential access to healthcare. Furthermore, although CUMC is not a referral center for NTM, it is a referral center for other conditions, including lung transplantation.

In conclusion, we found an increased incidence of NTM-positive cultures and disease compared with results in previous reports. Our results suggest that laboratory-based surveillance may produce reasonable estimates of the incidence of nonrespiratory tract disease and of disease caused by RGM, *M. kansasii,* and *M. marinum.* However, such surveillance is relatively inaccurate for estimating the incidence of pulmonary disease and disease caused by MAC. Larger, multicenter regional studies or mandatory reporting will be required to better understand the changing epidemiology of NTM in patients without HIV infection.
